# The Effect of Mixed Polymethylmethacrylate and Hydroxyapatite on Viability of Stem Cell from Human Exfoliated Deciduous Teeth and Osteoblast

**DOI:** 10.1055/s-0043-1768971

**Published:** 2023-06-19

**Authors:** Tania Saskianti, Shinta Purnamasari, Seno Pradopo, Alexander Patera Nugraha, Chiquita Prahasanti, Diah Savitri Ernawati, Masami Kanawa

**Affiliations:** 1 Department of Pediatric Dentistry, Faculty of Dental Medicine, Universitas Airlangga, Surabaya, Indonesia; 2 Department of Orthodontics, Faculty of Dental Medicine, Universitas Airlangga, Surabaya, Indonesia; 3 Department of Periodontics, Faculty of Dental Medicine, Universitas Airlangga, Surabaya, Indonesia; 4 Department of Oral Medicine, Faculty of Dental Medicine, Universitas Airlangga, Surabaya, Indonesia; 5 Natural Science Center for Basic Research and Development, Hiroshima University, Hiroshima, Japan

**Keywords:** viability, medicine, polymethylmethacrylate, hydroxyapatite, stem cell from human exfoliated deciduous teeth, osteoblast

## Abstract

**Objectives** Stem cell from human exfoliated deciduous teeth (SHED) has great potential for bone tissue engineering and cell therapy for regenerative medicine. It has been combined with biomaterials such as mixed of polymethylmethacrylate (PMMA) and hydroxyapatite (HA) as candidates for synthetic bone graft biomaterial. The aim of this study was to analyze the toxicity test of mixed PMMA-HA scaffold seeded with SHED and osteoblast *in vitro* .

**Materials and Methods** SHED was isolated from the pulp of noncarious deciduous teeth and osteoblast cells were cultured, and exposed to PMMA-HA scaffolds with three concentration groups: 20/80, 30/70, and 40/60 for 24 hours. Cytotoxicity test was performed by MTT assay to cell viability.

**Statistical Analysis** Data were analyzed using IBM SPSS Statistics 25, one-way analysis of variance followed by least significant difference test, considering the level of significance *p* -value less than 0.05

**Results** The percentage of SHED's viability was best in the PMMA-HA group with concentrations of 20/80, followed by 30/70, and 40/60 with 87.03, 75.33, and 65.79%, respectively. The percentage of osteoblast cell's viability was best in the PMMA-HA group with concentrations of 20/80, followed by 30/70, and 40/60 with 123.6, 108.36, and 93.48%, respectively.

**Conclusions** Mixed PMMA-HA was not toxic for the SHED and osteoblast. This characteristic is the initial requirement to be proposed as an alternative material for healing alveolar bone defects. In vivo animal research is mandatory to confirm the use of PMMA-HA on the alveolar defect model.

## Introduction

Periodontal disease, tooth loss, trauma, and infection are some of the factors that might cause alveolar bone defects. 1 In addition, congenital abnormalities in children such as cleft lip and palate (CLP) are also often accompanied by defects in the alveolar bone. CLP is one of the most common forms of congenital abnormalities with an incidence occurring in 1: 500 births in Asians and Native Americans and approximately 1 in 2,400 to 2,500 births in people of African. 2 3 Untreated alveolar bone defects usually lead to resorption of alveolar bone. The preservation of the alveolar ridge and the prevention of bone resorption are all achieved by filling these defects with bone graft material. 4 The gold standard bone graft material is autogenous bone because it has all the characteristics necessary for bone growth. 5 However, autogenous bone has some limitations, specifically bone availability and complications. Other bone graft materials, including xenograft, also have some disadvantages, such as the potential of xenogeneic bone blocks may crack during fixation, which could hinder the operation and the bone's healing process. 6

Polymethylmethacrylate (PMMA) is one type of polymer that is commonly used in dentistry and has been used as a fixation component in orthopaedic implants. 7 The flexible nature of PMMA makes it easy to manipulate the manufacture of biomaterials. The effort to strengthen the function of PMMA is by adding a bioactive ceramic material, namely hydroxyapatite (HA) that has excellent osteoconductive and osteointegration properties. 8 HA is a natural mineral form of calcium apatite and HA in bones is approximately 67 to 70% and has bioactive, biocompatible, and nontoxic properties. 9 Because HA is brittle, mixing it might be challenging; adding PMMA will provide mechanical structural integrity. The use of scaffolds with porous structures from bioceramic and polymeric components to support the growth of cells and bone tissue has been an attraction for a long time as an attractive candidate for biomaterials. 5 Tissue engineering techniques to replace missing or damaged functional tissues and organs with biomaterials that have good biocompatibility have developed rapidly. Research on stem cells has grown and both fields of medicine and dentistry have done substantial study of them. This prompted the researcher to apply the use of stem cells from human exfoliated deciduous teeth (SHED) from the human oral cavity with a scaffold derived from a mixture of natural biomaterials, namely mixed PMMA-HA. 6 10

Furthermore, whether this scaffold can be compatible with osteoblasts that are naturally present in the alveolar bone needs to be proven. Apart from being easy to obtain, SHED obtained from the pulp of primary children's teeth is an ideal source for bone regeneration because of its good viability and proliferative potential. SHED also showed positive results on osteogenic differentiation. 11 12 Many studies have demonstrated that SHEDs proliferate more quickly and have greater differentiation potential than bone marrow mesenchymal stem cells (BMSCs) or even Dental pulp stem cells (DPSCs). 13

Therefore, the selection of mixed PMMA-HA materials with SHED is considered because of their respective advantages that can complement each other as candidates for synthetic bone graft biomaterials. One of the important aspects in the initial screening and development of a mixed PMMA-HA as a candidate for synthetic bone graft biomaterials is the toxicity test, which aims to evaluate the toxicity and safety of these materials before interacting with the active ingredient, that is a SHED and osteoblast naturally present in the alveolar bone. According to the Telli et al 14 standard, it is stated that a substance is said to be nontoxic if the percentage of living cells after exposure to the substance is more than 50%. 15 We, therefore, hypothesize that the mixed PMMA-HA may not associated with toxicity of SHED and osteoblast.

Considering that the mixed PMMA-HA and its interaction with SHED and osteoblast are a new proposed biomaterial, to date there has been no research regarding it especially on the toxicity showed by cell's viability. The aim of this study was to analyze the toxicity test of mixed PMMA-HA scaffold seeded with SHED and osteoblast *in vitro* as a candidate of synthetic bone graft biomaterial.

## Materials and Methods

This was an experimental laboratory design research with a post-test-only control group design. The materials used in this study were PMMA (PMMA Granules ; HiMedia. Laboratories Pvt. Ltd. India), HA (Ceramic Center of the Ministry of Industry of the Republic of Indonesia), SHED obtained from the isolation of pulp tissue of noncarious primary teeth (Tissue Bank, Dr. Soetomo), 7F2 osteoblast (American Type Culture Collection, Manassas, Virginia, United States, CRL-12557), and MTT (Sigma Cat.No.M-5655).

### Mixed PMMA and HA Manufacturing

In this study, a preliminary test was performed to determine the ratio between groups. This comparison is based on the HA content in the bones. The procedure for making mixed PMMA-HA scaffold was performed by weighing PMMA and HA scaffold was done by weighing 1 g of PMMA, 2 mL of acetone, and 4 g of HA powder for a 20:80 ratio; 1.5 g of PMMA, 3 mL of acetone, and 3.5 g of HA powder for a 30:70 ratio; 2 g of PMMA, 4 mL of acetone, and 3 g of HA powder for a 40:60 ratio, respectively.

**Fig. 1:**
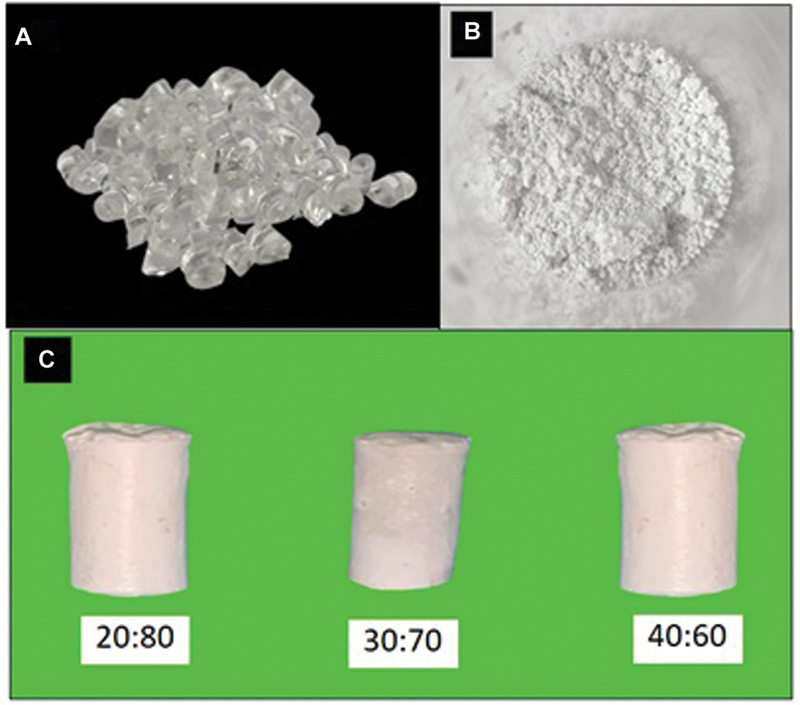
Mixed polymethylmethacrylate and hydroxyapatite material. ( **A** ) Polymethylmethacrylate granules. ( **B** ) Hydroxyapatite from Balai Besar Keramik Indonesia. ( **C** ) Three groups of mixed polymethyl methacrylate and hydroxyapatite.

PMMA that has been weighed was put into a bottle and mixed with acetone, then stirred briefly until the PMMA grains were submerged in acetone, then left in the refrigerator at a temperature of −30° for 24 hours. After 24 hours, the HA powder was added to the PMMA solution and then stirred using a spatula over a magnetic stirrer until it became homogeneous. After the PMMA:HA mixture became homogeneous, it was poured into a mold with 5 mm of diameter and height as shown in Fig. 1 . 16

After that, a freeze-drying process was performed. The mixed PMMA and HA that had been freeze-dried was subjected to gamma radiation sterilization at the Indonesian Nuclear Energy Agency (BATAN).

### Isolation and Culture of Stem Cells from Human Exfoliated Deciduous Teeth

The SHED was collected from deciduous teeth using the following criteria: #73 persistence of deciduous tooth, free of cavities, no root resorption, and a vital and undamaged pulp was retrieved after tooth extraction from a healthy, 8-year-old pediatric patient. Patient anonymity was maintained and written informed consent was obtained from the patient's parents. The ethical clearance was approved by the Ethical Research Committee Faculty of Medicine Universitas Airlangga no. 239/HRECC. FODM/V/2021 covered for human sampling.

**Fig. 2:**
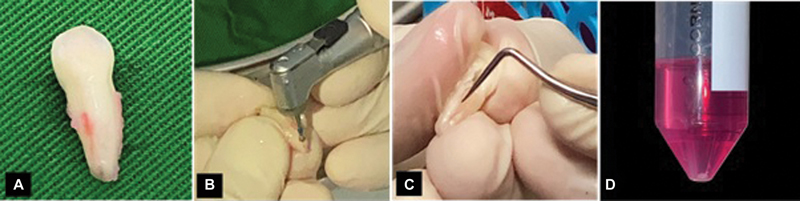
Stem cell from human exfoliated deciduous teeth isolation protocol. ( **A** ) #73 persistence of deciduous tooth, free of cavities, and no root resorption. ( **B–C** ) The dental pulp cavity was opened using drills with sterilized round bur. ( **D** ) Pulp tissue was taken in the medium within a 15 ml conical tube.

The pulp tissue was placed in the medium within a 15 mL conical tube and placed in a cool box to be immediately sent to the GDC Tissue Bank Dr. Soetomo Surabaya ( Fig. 2 ). Then the cells were cultured in Dulbecco's Modified Eagle Medium (DMEM, Life Technologies, Gibco BRL, United States) with the addition of 20% fetal bovine serum (FBS, Biochrom AG, Germany), 5 mL L-glutamine (Gibco Invitrogen, United States), 100 U/mL penicillin-G, 100 g/mL streptomycin, and 100 g/mL kanamycin (Gibco Invitrogen, 25, United States). 17

**Fig. 3:**
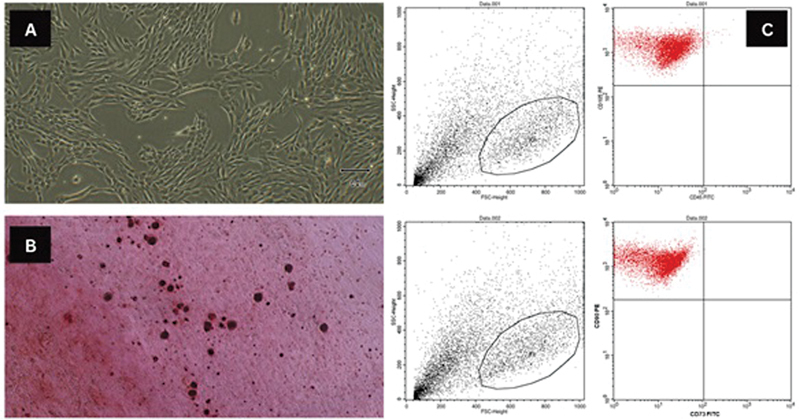
Characterization of stem cell from human exfoliated deciduous teeth **.** ( **A** ) Morphology of isolated and unstained MSCs demonstrating a typical mesenchymal stem cell shape characterization of adherent spindle-shaped MSCs cells in culture. ( **B** ) Flow cytometry analysis of passage 3 mesenchymal stem cells culture for CD105, CD90, CD45, and CD73cells. ( **C** ) Calcium deposition can be seen after staining with Alizarin Red in the osteogenic test culture.

After 3 days, the medium was discarded to take off the portion of the cell that was not connected to the plate and place it in a new medium. At this stage, fibroblast growth factor-2 was added. After the cells were confluent, they were passaged using 0.05% trypsin-ethylenediamine tetraacetic acid (EDTA) and after that the cells were washed and cultured again in 60- or 100-mm tissue culture dishes (Corning). After the confluent cells are repassed, and the cells can be used for research ( Fig. 3A ). 17

### Characterization of Stem Cells from Human Exfoliated Deciduous Teeth

SHEDs were washed twice with phosphate buffered saline (PBS) containing 2% fetal bovine serum (Gibco; Thermo Fisher Scientific, Inc.) and were incubated for 30 minutes at room temperature with antibodies against CD45-FITC, CD73-FITC, CD90-PE, and CD105-PE. All antibodies were used at a dilution of 1:100 and purchased from BD Biosciences. Samples were diluted up to 1 μL and read with flow cytometry (FACS Callibur, BD).

### Osteogenic Differentiation (Alizarin Red Staining)

SHED was induced for osteogenic differentiation (OsteoMAX-XFTM Differentiation Medium) according to the manufacturer's instruction. Briefly, 2 × 10 ^4^ cells per well were added in a 48-well plate and grown until confluence. Confluent cells of 0.5 mL OsteoMAX-XFTM differentiation medium were added to each well. This medium change corresponds to differentiation day 1. On day 3, 0.25 mL of the medium was removed from each well and replaced it with 0.5 mL of fresh OsteoMAX-XFTM differentiation medium. For all subsequent medium changes, 0.5 mL of the medium was removed from each well and replaced it with 0.5 mL of fresh OsteoMAX-XFTM differentiation medium. Medium changes should occur every 3 days for 14 to 17 days. After 14 to 17 days of differentiation, osteocytes could be fixed and stained for alkaline phosphatase (Cat. No. SCR004) or with Alizarin Red (Cat. No. ECM815) for mineralization.

### Osteoblast Cell Culture

7F2 osteoblast (American Type Culture Collection, Manassas, VA, United States, CRL-12557) was cultured in DMEM media supplemented with 10% fetal bovine serum and streptomycin penicillin. Cells were cultured in 75 cm ^2^ flasks and allowed to grow until confluent. The cultures were incubated at 37°C with 5% CO _2_ and the culture medium was changed every 48 to 18

The cleaned media was rinsed with PBS and then added 1 to 2 mL of the trypsin–EDTA solution. Flask was left at 37°C incubators until the cells were released. The cell suspension was then centrifuged at 2000 rpm for 10 minutes, pelleted the suspension back into the new medium, aspirated, put into a new flask, and then subcultured before the cells became confluent. The osteoblast cells were counted using hemocytometer and seeded in 96 well plates with a concentration of 2 × 10 ^5^ /well. 18

### MTT Assay

Cells viability was evaluated by cytotoxicity test using MTT assay. SHED at passages 4 to 5 and osteoblast cells at passages 4 were prepared 80% confluent. There were two big groups in this study; each group consisted four groups. There were four groups in this study, control group (without PMMA/ HA scaffold), group 1 (with 20/80 PMMA-HA scaffold), group 2 (with 30/70 PMMA-HA scaffold), and group 3 (with 40/60 PMMA-HA scaffold). Five repetitions were performed in each group so that the total sample was 40 scaffolds. The cells were harvested until become single cells and homogenized in the culture medium. The cells were planted in 96 well plates with a concentration of 2 × 10 ^5^ /well and the empty wells were left blank. CO _2_ was incubated in an incubator for 24 hours until the cells adhered perfectly.

The test material was prepared in the form of a mixture of PMMA and HA with concentration groups of 20/80, 30/70, and 40/60, and immersed in the culture medium. Soaking medium of 100 µL was added for each type of test material into the well. It was incubated again for another 24 hours. A total of 25 µL MTT was added to each well and it was incubated again for 4 hours. Then the medium and MTT were discarded. dimethyl sulfoxide (DMSO) of 200 µL was added to each well, and when the color changed into purple, then 200 µL of PBS was added to an empty well and inserted into the enzyme-linked immunosorbent assay (ELISA) reader.

The absorbance of each well was read at a wavelength of 595 nm. The cytotoxicity is expressed as cytotoxic dose, the concentration of the substance inhibiting cell growth by 50% (CD50). 19

### Statistical Analysis

Statistical analysis was performed using IBM SPSS Statistics Software, version 25.0 (IBM Corp., Armonk, New York, United States). The data were statistically analyzed by using one-way analysis of variance (ANOVA) followed by least significant difference (LSD) test, considering the level of significance *p* -value less than 0.05.

## Results

### Isolation, Culture, and Characterization of Stem Cells from Human Exfoliated Deciduous Teeth

SHEDs were cultured in nonosteogenic culture media. SHEDs were subcultured until it reaches passage 3. All adherent cells showed spindle-shaped morphology under electron microscope ( Fig. 3A ). SHEDs within three passages were shown to be MSCs by flow cytometry. SHEDs expressed the mesenchymal stem cell surface markers (CD90, and CD105), but were negative for CD45 and CD73 markers ( Fig. 3B ). Osteogenic potential differentiation of SHED was confirmed by the presence of calcium deposits on Alizarin Red S staining on day 14 ( Fig. 3C ).

### SHED and Osteoblast Viability

**Fig. 4:**
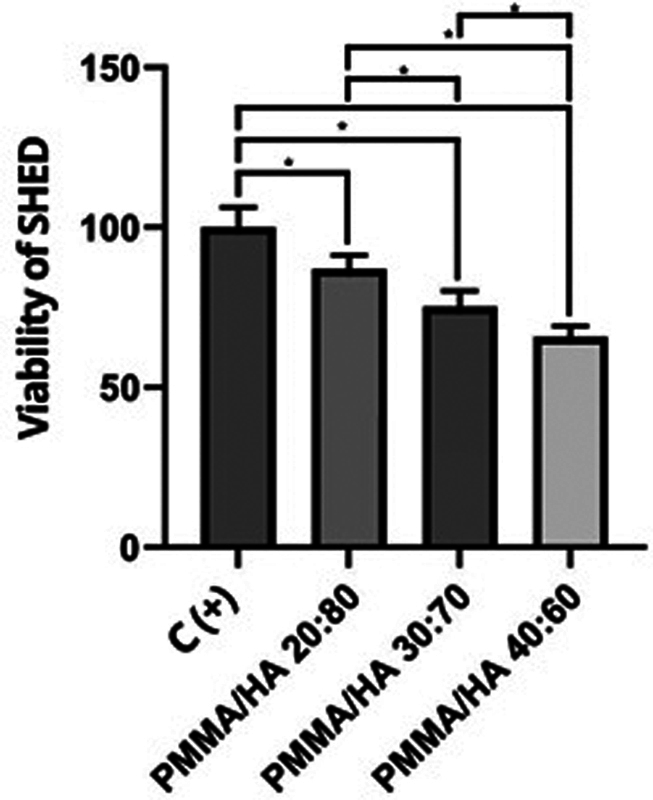
Effects of different mixed polymethylmethacrylate and hydroxyapatite (PMMA-HA) on viability of stem cell from human exfoliated deciduous teeth (SHED **)** . Statistically significant ( *p* < 0.05; least significant difference test differences in values compared with the control value (untreated) are indicated by an SPSS. Graphics represent the means and ± standard deviation from three independent determinations performed in five replicates.

**Fig. 5:**
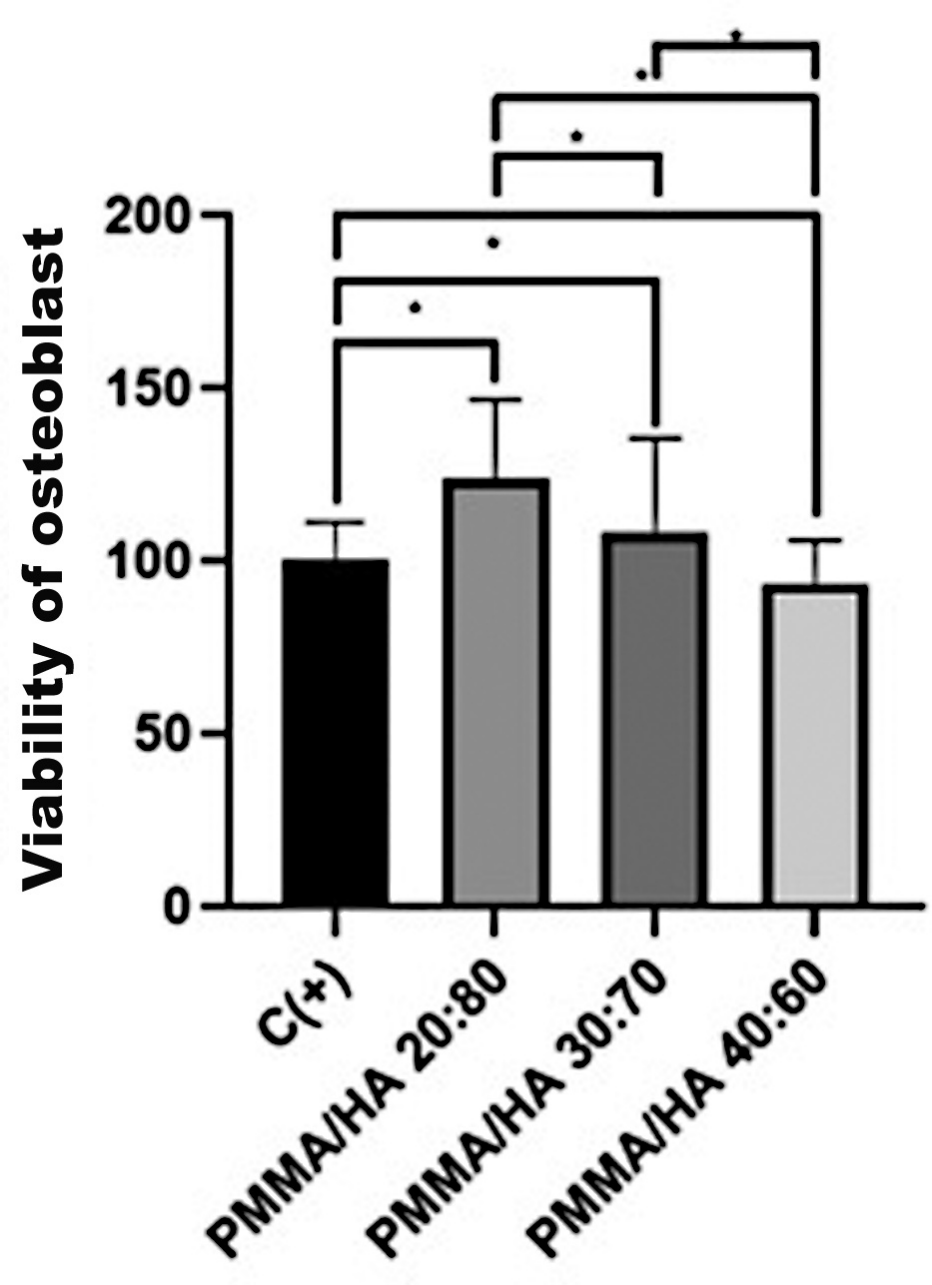
Effects of different mixed polymethylmethacrylate and hydroxyapatite (PMMA-HA) on viability of osteoblast. Statistically significant ( *p* < 0.05; least significant difference) test differences in values compared with the control value (untreated) are indicated by an SPSS. Graphics represent the means and ± standard deviation from three independent determinations performed in five replicates.

The cytotoxicity effect of mixed PMMA-HA on SHED and osteoblast that represent cells viability were assessed using the MTT assay method based on the absorbance value detected by ELISA reader to see the number of cells in optical density units and converted into the cell viability formula. The percentage of viability of SHED and osteoblast cells against the mixed PMMA-HA can be seen in Figs. 4 and 5 . Fig. 4 showed the 20/80 groups has the highest mean percentage of SHED's viability of 87.03% and the percentage of osteoblast's viability of 123.6%. The data obtained were homogeneous and normally distributed, tested with the one-sample Kolmogorov–Smirnov test. To determine the difference in viability of SHED and osteoblast against the mixed PMMA-HA, statistical calculations were performed using one-way ANOVA, obtained *p* -value less than 0.05. The results of this analysis showed that there was a significant difference in the viability of SHED and osteoblast against the mixed PMMA-HA. LSD analysis showed significant difference between each group of mixed PMMA-HA.

## Discussion

The purpose of tissue engineering is to develop tissue reconstruction that is useful for restoring, maintaining, repairing, or enhancing the function of tissues that are damaged or lost due to physiological, pathological, and mechanical conditions or trauma. 20 Three important components in tissue engineering are stem cells/progenitor cells, signaling, and scaffold. 15 21 These three important components are known as the tissue engineering triad because they are arranged in such a way that they resemble the natural regeneration that occurs in cells, tissues, and organs. 22 Scaffold is a porous solid biomaterial with a three-dimensional shape that was designed to deliver sufficient nutrients, gases, and regulatory factors to allow interactions between cells and biomaterials, cell adhesion, and extracellular matrix deposits to decay at a controlled rate according to the rate at which the material was deposited, tissue regeneration and minimize inflammatory reactions. 23 Scaffold can be made from synthetic bone graft material that has the ability to induce bone formation. One of the ideal requirements of synthetic bone graft is that it is biocompatible or not toxic. 19 24

Cytotoxicity refers to cell damage, where cells can die due to necrosis or apoptosis (programmed cell death). 20 25 Cytotoxicity test is a method to determine whether a substance is toxic to certain cells. The parameter of toxicity test is cell viability. One of the methods to assess the cytotoxicity of a substance is by an enzymatic test with MTT assay reagent. This method was chosen because it has good sensitivity in evaluating the cytotoxicity of the test material. 26 It also has a relatively fast procedure step and is easy to retest when needed. This method measures the metabolic activity of cell growth after exposure to the material test. The basic principle is to measure cellular activity based on the activity of the enzyme succinate dehydrogenase in the mitochondria of cells to reduce the tetrazolium salt MTT. This enzyme will react with MTT and form purple formazan crystals whose amount is proportional to the activity of living cells because these crystals are impermeable to dead cell membranes. 26 The parameter used for the cytotoxic test is the IC _50_ value (50% inhibition concentration). The IC _50_ value is a concentration value that indicates the inhibition of cell proliferation by 50% and the potential toxicity of material to cells. IC _50_ value can indicate the potential of the material as cytotoxic. This value is a benchmark for conducting a cell kinetics observation test. The greater the IC _50_ value, the lower toxicity of the material to cells. 27

PMMA-HA cytotoxicity check was performed on SHED and osteoblast cultures as a candidate of synthetic bone graft materials that is expected to regenerate bone in alveolar defects. This is based on the role of SHED and osteoblast as active cells responsible for osteogenic differentiation and bone matrix formation. 11 Based on Su Min Lee's research, 28 which considered SHED's potent osteogenic potential as well as its successful use in the regeneration of teeth and the bone regeneration of the craniofacial region. In addition, SHED is one of the mesenchymal stem cells that has a high level of sensitivity to toxic agents so it is often used in toxicity tests. In this study, SHED expressed the mesenchymal stem cell surface markers (CD90, and CD105), but was negative for CD45 and CD73 markers. The nonuniform expression of CD73 on MSC may be associated with the reparative property, as extracellular adenosine catalyzed by the dephosphorylation activity of CD73 has been proven as a pivotal regulator of local immune responses. 29

Quantitative data from the research was obtained by measuring the percentage of the number of living cells in each group. The results of this study showed that all treatment groups did not have toxic properties against SHED and osteoblast according to the Telli et al 11 standard, which states that a substance is said to be nontoxic if the percentage of living cells after exposure to the substance is more than 50%. The results of cell viability that have been exposed to a mixed PMMA-HA for 24 hours with several concentrations, cell viability of SHED ranged from 65.79 to 87.03% and cell viability of osteoblast ranged from 93.48 to 123,6%; so it can be said to be nontoxic. These results were consistent with Pridanti et al 30 on umbilical cord mesenchymal stem cells at all concentrations due to high Ca/P ratio on HA. Similar result was shown by Gayathri et al's 31 with adipose-derived mesenchymal stem cells. Additionally, Mostafa's research, which evaluated the PMMA-HA-MgP nanocomposite combination's compression strength and cytotoxicity against fibroblast cells, revealed that the HA ratio group with a weight ratio of 7.5% and a weight ratio of 6.12% had the highest compression strength and cell viability values.

The combination of PMMA-HA did not have a toxic effect because the HA content in mixed PMMA-HA was directly proportional to the percentage of SHED viability. HA is an inorganic material that contained approximately 67 to 70% of the bone. HA has good biocompatibility and bioactivity properties. 10 22 The elements present in HA are dominated by elements of calcium (Ca), oxygen (O), phosphorus (P), and other with minimal amount elements (<5%), namely aluminum (Al), silica (Si), sodium (Na), and magnesium (Mg). 16 32 Ca contributes to the signaling of osteoblast growth and extracellular matrix. The high amount of calcium would increase the proliferation, due to the increase of calcium channel expression. Calcium sensing receptor could detect any external Ca ^2+^ concentration change and increase Ca ^2+^ influx. The higher influx in higher calcium ions concentration would induce cellular responses such as cell proliferation. 30 The increase in extracellular Ca ^2+^ induced expressions of cell growth factors (fibroblast growth factor 2 and transforming growth factor beta 1) and the levels of cell cycle regulators. Therefore, we expected that these factors might mediate the increase extracellular Ca ^2+^ -induced cell proliferation. 33 A calcium channel overexpression might be inhibited by an excessive concentration of extracellular Ca ^2+^ ions. Calcium phosphate concentrations would rise as Ca ^2+^ ions moved into the cytoplasm. This increase would cause the endoplasmic reticulum to produce intracellular Ca ^2+^ ions, which would disrupt intracellular Ca ^2+^ homeostasis and act as a secondary messenger in preserving cell function, predisposing to mitochondrial-mediated apoptosis. 34

Phosphorus (P) will regulate the signal of proliferation, differentiation, mineralization of osteoblasts, and apoptosis of osteoclasts. 24 35 When cells are exposed to high quantities of phosphate, osteoblast apoptosis can be triggered. This could be due to the mitochondrial membrane being severely damaged. 36 Si stimulates DNA synthesis and cell growth in fibroblast and bone cells from mammals. Si ions stimulate osteogenesis via raising levels of alkaline phosphatase (ALP) and osteocalcin, dentin sialoprotein (DSP), and mineralization as well as increased proliferation states. It has been well documented that silica may modify biological responses thus supporting stem cells and growth factors in the tissue engineering process. 37 Furthermore, the amount of Al and Mg in HA may induce cells apoptosis, which is determined by dose and duration. Changes in the core morphology indicate that Al _2_ O _3_ nanoparticles alter cell cycle progression and gene expression. Corrosion products of Mg can significantly affect metabolic activity and cell proliferation, which in turn affects cell fusion/differentiation. 38 39 However, because the content of Al and Mg in HA is below 5 wt%, it is not expected to have a toxic effect on cells.

Unlike HA, PMMA has a different nature. PMMA is composed of the elements carbon (C), oxygen (O), and hydrogen (H), which contribute to the mechanical stability of the bone graft by increasing the scaffold's structural integrity. 40 The above elements have a positive impact on the viability of SHED and osteoblast. However, this study was limited to the cytotoxicity test of the mixed PMMA-HA against SHED and osteoblasts.

## Conclusion

According to the research, it was concluded that mixed PMMA-HA was not toxic for the SHED and osteoblast. This characteristic is the initial requirement to be proposed as an alternative material for healing alveolar bone defects. Further research studying the proliferation rate of SHED and osteoblasts after implanted in mixed PMMA-HA is needed. *In vivo* animal research is mandatory to confirm the use of PMMA-HA on the alveolar defect model.

## References

[1] Gokmenoglu C Yavuzl M C Sadik E Treatment of different types of bone defects with concentrated growth factor: four case reports Int J Dent Oral Health 2016 2 29

[2] Shah S Ali S Sahito M Evaluating occurrence of variable cleft lip and palate types among ethnic groups of Malaysia J Pak Dent Assoc 2018 27 9 12

[3] Yilmaz R BN Cakan D G Mesgarzadeh N Prevalence and management of natal/neonatal teeth in cleft lip and palate patients Eur J Dent 2016 10 01 54 58 27011740 10.4103/1305-7456.175698 PMC4784154

[4] Kumar P Vinitha B Fathima G Bone grafts in dentistry J Pharm Bioallied Sci 2013 5 01 S125 S127 23946565 10.4103/0975-7406.113312 PMC3722694

[5] Bahtiar R Kurniasih D H Alifianto U The difference of bovine bone graft and iliac crest bone graft effect on closure of bone defect in alveolar bone graft in Dr. Moewardi Hospital and Panti Waluyo Hospitals, Surakarta Indones J Med 2018 3 03 151 161

[6] Tomas M Čandrlić M Juzbašić M Synthetic injectable biomaterials for alveolar bone regeneration in animal and human studies Materials (Basel) 2021 14 11 2858 34073551 10.3390/ma14112858 PMC8197881

[7] Teo A JT Mishra A Park I Kim Y J Park W T Yoon Y J Polymeric biomaterials for medical implants and devices ACS Biomater Sci Eng 2016 2 04 454 472 33465850 10.1021/acsbiomaterials.5b00429

[8] Gomes D S Santos A M Neves G A Menezes R R A brief review on hydroxyapatite production and use in biomedicine Ceramica 2019 65 282 302

[9] Habibie S Tristiyanti Y Gustiono D Harahap M E Chalid S Y Effendi D Production and characterization of scaffold made of hydroxyapatite and pectin of Green Cincau Leaf (Premna oblongifolia Merr) J Eng Res 2019 1 01 12 16

[10] Vajrabhaya L O Korsuwannawong S Surarit R Cytotoxic and the proliferative effect of cuttlefish bone on MC3T3-E1 osteoblast cell line Eur J Dent 2017 11 04 503 507 29279678 10.4103/ejd.ejd_159_17 PMC5727737

[11] Saskianti T Ramadhani R Budipramana E S Pradopo S Suardita K Potential proliferation of stem cell from human exfoliated deciduous teeth (SHED) in carbonate apatite and hydroxyapatite scaffold J Int Dent Med Res 2017 10 02 350 353

[12] Puspitasari T W Saskianti T Tedjosasongko U Karakterisasi stem cell pulpa gigi sulung dengan modifikasi enzim tripsin (The characterization of stem cells from human exfoliated deciduous teeth using trypsin enzyme) Dent J 2014 47 02 115 119

[13] Jindal L Stem cells from human exfoliated deciduous teeth (SHED) – turning useless into miracle: a review article Acta Scientific Dental Sciences 2019 3 10 49 54

[14] Telli C Serper A Dogan A L Guc D Evaluation of the cytotoxicity of calcium phosphate root canal sealers by MTT assay J Endod 1999 25 12 811 813 10726527 10.1016/S0099-2399(99)80303-3

[15] Hussain T Garg T Goyal A K Rath G Biomedical applications of nanofiber scaffolds in tissue engineering J Biomater Tissue Eng 2014 4 08 600 623

[16] Saskianti T Novianti A Sahar D Mixed polymethylmethacrylate and hydroxyapatite as a candidate of synthetic graft materials for cleft palate J Int Dent Med Res 2022 15 02 538 543

[17] Saskianti T Nugraha A P Prahasanti C Ernawati D S Suardita K Riawan W Immunohistochemical analysis of stem cells from human exfoliated deciduous teeth seeded in carbonate apatite scaffold for the alveolar bone defect in Wistar rats ( *Rattus novergicus* ) F1000 Res 2020 9 1164 10.12688/f1000research.25009.1 PMC7721066 33335716

[18] Braveboy-Wagner J Lelkes P I Impairment of 7F2 osteoblast function by simulated partial gravity in a Random Positioning Machine NPJ Microgravity 2022 8 01 20 35672327 10.1038/s41526-022-00202-x PMC9174291

[19] Silva S L Chaar J D Figueiredo P D Yano T Cytotoxic evaluation of essential oil from Casearia sylvestris Sw on human cancer cells and erythrocytes Acta Amazon 2008 38 107 112

[20] Caddeo S Boffito M Sartori S Tissue engineering approaches in the design of healthy and pathological *in vitro* tissue models Front Bioeng Biotechnol 2017 5 40 28798911 10.3389/fbioe.2017.00040 PMC5526851

[21] Anbu R T Suresh V Gounder R Kannan A Comparison of the efficacy of three different bone regeneration materials: an animal study Eur J Dent 2019 13 01 22 28 31170752 10.1055/s-0039-1688735 PMC6635883

[22] Herda E Puspitasari D Overview of the role and properties of the material used as a scaffold in tissue engineering J Material Ked Gigi 2018 5 01 56 63

[23] Kartikasari N Yuliati A Kriswandini I L Compressive strength and porosity tests on bovine hydroxyapatite-gelatin-chitosan scaffolds Dent J 2016 49 03 153 157

[24] Alaribe F N Manoto S L Motaung S C Scaffolds from biomaterials: advantages and limitations in bone and tissue engineering Biologia 2016 71 04 353 366

[25] Elshahawy W Cytotoxicity of dental ceramics used for manufacturing dental fixed prosthesis: a systematic review M J Dent 2016 1 02 10

[26] Doyle A Griffith S JB Cell and Tissue Culture for Medical Research 49th ed. New York John Willey and Sons 2000

[27] Haryoto H Muhtadi M Indrayudha P Azizah T Suhendi A Activity of sitotoksik ethanol extract plant growth (Cynometra ramiflora Linn) on heavy cell, T47D and WiDR J Penel Sain 2013 18 02 21 28

[28] Lee S M Zhang Q Le A D Dental stem cells: sources and potential applications Curr Oral Health Rep 2014 1 01 34 42

[29] Tan K Zhu H Zhang J CD73 expression on mesenchymal stem cells dictates the reparative properties via its anti-inflammatory activity Stem Cells Int 2019 2019 8.717694E6 10.1155/2019/8717694 PMC6525959 31249602

[30] Pridanti K A Cahyaraeni F Harijanto E Characteristics and cytotoxicity of hydroxyapatite from Padalarang–Cirebon limestone as bone grafting candidate Biochem Cell Arch 2020 20 02 4727 4731

[31] Gayathri V Harikrishnan V Mohanan P V Integration of rabbit adipose derived mesenchymal stem cells to hydroxyapatite burr hole button device for bone interface regeneration Int J Biomater 2016 2016 1.067857E6 10.1155/2016/1067857 PMC4736387 26880922

[32] Margareta M AH Fuad A Ilmiawati S A Wonorahardjo S Synthesis of hydroxyapatite Ca _10_ (PO _4_ ) _6_ (OH) _2_ based on lime stone Jurnal Penelitian Fisika dan Aplikasinya 2015 5 15 20

[33] Lee M N Hwang H S Oh S H Elevated extracellular calcium ions promote proliferation and migration of mesenchymal stem cells via increasing osteopontin expression Exp Mol Med 2018 50 11 1 16 10.1038/s12276-018-0170-6 PMC6215840 30393382

[34] Han Y High concentrations of calcium suppress osteogenic differentiation of human periodontal ligament stem cells in vitro J Dent Sci 2021 16 03 817 824 34141094 10.1016/j.jds.2021.02.011 PMC8189895

[35] Jeong J Kim J H Shim J H Hwang N S Heo C Y Bioactive calcium phosphate materials and applications in bone regeneration Biomater Res 2019 23 01 4 30675377 10.1186/s40824-018-0149-3 PMC6332599

[36] Alsenan J Chou L Inorganic phosphate effect of on human dental pulp cell cultures Int J Mater Sci Eng App 2019 8 40 46

[37] Alsenan J Effect of silicon and calcium on human dental pulp cell cultures Int J Mater Sci Eng App 2017 6 290

[38] Maradze D Musson D Zheng Y Cornish J Lewis M Liu Y High magnesium corrosion rate has an effect on osteoclast and mesenchymal stem cell role during bone remodelling Sci Rep 2018 8 01 10003 29968794 10.1038/s41598-018-28476-w PMC6030161

[39] Pallavi M Waterman J Koo Y Sankar J Yun Y Assessment of cytotoxicity of magnesium oxide and magnesium hydroxide nanoparticles using the electric cell-substrate impedance sensing App Sci 2020 10 06 2114

[40] Oryan A Alidadi S Bigham-Sadegh A Moshiri A Healing potentials of polymethylmethacrylate bone cement combined with platelet gel in the critical-sized radial bone defect of rats PLoS One 2018 13 04 e0194751 29608574 10.1371/journal.pone.0194751 PMC5880368

